# Clinical outcomes and potential therapies prediction of subgroups based on a ferroptosis-related long non-coding RNA signature for gastric cancer

**DOI:** 10.18632/aging.204227

**Published:** 2022-08-14

**Authors:** Haigang Geng, Ruolan Qian, Linmeng Zhang, Chen Yang, Xiang Xia, Cun Wang, Gang Zhao, Zizhen Zhang, Chunchao Zhu

**Affiliations:** 1 Department of Gastrointestinal Surgery, Renji Hospital, School of Medicine, Shanghai Jiao Tong University, Shanghai, China; 2 State Key Laboratory of Oncogenes and Related Genes, Shanghai Cancer Institute, Renji Hospital, Shanghai Jiao Tong University School of Medicine, Shanghai, China

**Keywords:** ferroptosis, lncRNA, gastric cancer, immune, targeted therapies

## Abstract

Background: Gastric cancer (GC) is one of the most aggressive malignant tumors worldwide. Ferroptosis is a kind of iron-dependent cell death, which is proved to be closely related to tumor progression. In this study, we aim at constructing a ferroptosis-related lncRNAs signature to predict the prognosis of GC and explore potential therapies.

Methods: Ferroptosis-Related LncRNAs Signature for GC patients (FRLSG) was constructed through univariate Cox regression, the LASSO algorithm, and multivariate Cox regression. Kaplan–Meier analysis, receiver operating characteristic curves, and risk score plot were applied to verify the predictive power of FRLSG. Gene Set Enrichment Analysis (GSEA) and immune infiltration analyses were conducted to explore the potential clinical value of the FRLSG. In addition, drug sensitivity prediction was applied to identify chemotherapeutic drugs with potential therapeutic effect.

Results: Five ferroptosis-related lncRNAs (AC004816.1, AC005532.1, LINC01357, AL355574.1 and AL049840.4) were identified to construct FRLSG, whose expression level in GC were confirmed by experimental validation. Kaplan-Meier curve and ROC curve proved the reliability and effectiveness of the FRLSG in predicting the prognosis for GC patients. Several immune-related pathways were enriched in the high-FRLSG group, and further immune infiltration analyses demonstrated the high immune infiltration status of the high-FRLSG group. In addition, 19 and 24 candidate drugs with potential therapeutic effect were identified for the high- and low-FRLSG groups, respectively.

Conclusions: FRLSG was an effective tool in predicting the prognosis of GC, which might help to prioritize potential therapeutics for GC patients.

## INTRODUCTION

Gastric cancer (GC), as the fifth most common malignancy, leads to the fourth-highest cancer death worldwide [1]. Over one million people are diagnosed with GC every year, which entails a major burden on mobility, mortality, comorbidities, and costs [2]. Although clinical therapy choices such as surgery and systemic chemotherapy advance continuously, the prognosis of advanced GC patients is still very poor [3]. Up to now, traditional evaluation indicators, such as Lauren/WHO classification and tumor-node-metastasis (TNM) staging, are the main methods to evaluate the prognosis of GC patients [4], while the prognostic prediction ability of these indicators is always limited due to the uncharacterized genetic alterations. Therefore, a novel biomarker with reliability and effectiveness is needed for the clinical treatment of GC patients.

Long non-coding RNAs (lncRNAs) are defined as non-coding RNAs with at least 200 bp in length, which are proved to be key regulators in the occurrence and development of malignancies [5, 6]. Aberrant expression of lncRNAs could affect the initiation and progression in a spectrum of malignancies, such as lung cancer [7], breast cancer [8] and colon cancer [9]. Meanwhile, many studies have demonstrated that abnormal expression of lncRNAs like MALAT1 [10], ARHGAP27P1 [11], PANDAR [12] and PTAR [13] would affect the development and progression of GC. In addition, competing endogenous RNAs (ceRNAs) network composed of lncRNAs and mRNAs could influence drug response and contribute to drug resistance in cancer therapy [14]. Currently, the prognosis evaluation of cancer patients receives more and more attention, because it directly affects the choice of treatment to a certain extent. Compared with traditional standard for evaluating cancer prognosis, lncRNA-based signatures demonstrate higher predictive accuracy and better universality, which are increasingly used in clinical work [15].

The past decade has witnessed an outbreak of ferroptosis-associated researches. Ferroptosis, featured in intracellular accumulation of the massive lipid peroxidation, is a kind of regulated necrosis in iron-dependent form, which is distinct from apoptosis, necroptosis, and autophagy [16]. Excessive or defective ferroptosis is proved to be a risk factor for promoting tumorigenesis and as well accelerating malignant processes [17]. Meanwhile, ferroptosis could induce inflammation and immunity response, which would affect cancer progression [18]. For example, Wang et al. [19] found that CD8+ T cells activated by cancer immunotherapy could promote tumor cell lipid peroxidation and ferroptosis, thus contributing to the potential anti-tumor approach of immunotherapy. Several drugs, such as sorafenib, statins and artemisinin, might exert therapeutic effects by inducing ferroptosis [20]. Sun et al. [21] reported that the compound extracted from Chinese liverworts Jungermannia tetragona Lindenb could induce apoptosis and ferroptosis to sensitize cancer cells which were resistant to cisplatin.

Previous studies reported that specific lncRNAs could induce or inhibit ferroptosis in GC cells under different circumstance. LncRNA-PMAN was upregulated in GC cells and inhibited Erastin- and RSL3-induced ferroptosis, leading to poor prognosis and peritoneal metastasis [22]. A hypoxia-induced lncRNA-CBSLR could protect GC cells from ferroptosis, which could contribute to chem-resistance in GC patients [23]. Considering the tight relationship among ferroptosis, lncRNA, and GC, we constructed a molecular signature based on the ferroptosis-related lncRNAs (FRLs) for GC patients in this study, aiming to help the prognosis prediction of GC.

## METHODS

### Data collection

Transcriptome data of GC patients and corresponding clinical information were derived from the TCGA data portal (https://portal.gdc.cancer.gov/, accessed on Aug 3, 2021), and a total of 375 GC samples and 32 non-tumor tissues were obtained. Samples without complete clinical data and the OS < 30 days were extracted to maintain statistical power and reduce bias. Ultimately, a total of 334 GC patients were included and utilized for subsequent analyses.

### Identification of FRLs

The list of ferroptosis-related genes (FRGs) was obtained from FerrDb [24], which was an online database containing a comprehensive list of genes and proteins associated with ferroptosis. Differentially expressed lncRNAs (| log2 fold change (FC) | > 1 and FDR < 0.05) between the GC tissue and normal tissue were identified with “limma” R package. Then Pearson correlation analysis was applied to assess the relationship between the FRGs and differentially expressed lncRNAs. LncRNAs with correlation coefficient R^2^ > 0.3 and *P* value < 0.001 were considered to be tightly associated with ferroptosis, which were regarded as FRLs.

### Construction of FRLSG and evaluation of prognosis prediction ability

First, identified FRLs were subjected to univariable Cox regression to select the lncRNAs associated with prognosis of GC patients. Then these prognostic FRLs were further screened using the LASSO penalty analysis located in the “glmnet” package in R [25]. Finally, multivariable Cox regression analysis was conducted to construct the Ferroptosis-Related LncRNAs Signature for GC patients (FRLSG). The formula of FRLSG was as follows:

$$\begin{array}{c} FRLSG=(Coefficient\;FRL1\times expression\;of\;FRL1)+(Coefficient\;FRL2\times expression\;of\;FRL2)\\ +\cdots +(Coefficient\;FRLn\times expression\;of\;FRLn). \end{array}$$

GC patients were separated into the high- and low-FRLSG groups based on the median FRLSG score. Kaplan–Meier curve analysis was conducted to assess the survival difference between the high- and low-FRLSG groups with Log-rank *P* test. The sensitivity and specificity of the FRLSG in predicting prognosis were compared with other clinical features using the receiver operating characteristic (ROC) analysis, and the result was visualized by the “survivalROC” R package. Moreover, principal component analysis (PCA) and t-distribution random neighbor embedding (t-SNE) were conducted based on five FRLs using R packages “stats” and “Rtsne”, respectively.

### Construction of the FRLSG-integrated nomogram and gene set enrichment analysis

Univariate and multivariate analyses were conducted on FRLSG and clinicopathological manifestations for the identification of independent prognostic factors. Based on the identified independent prognostic factors, the FRLSG-integrated nomogram was constructed for predicting of 1/3/5-year overall survival of GC patients, which was validated by the calibration curves. Gene Set Enrichment Analysis (GSEA) was applied to detect the different functional phenotypes between the low-FRLSG subgroup and high-FRLSG subgroup in the enrichment of pathways in Kyoto Encyclopedia of Genes and Genomes (KEGG). 1000 random gene set permutations were performed. In addition, Gene Set Variation Analysis (GSVA) was performed to investigate the difference of enrichment score in certain pathway between the high- and low-FRLSG groups.

### Immune infiltration analyses

With CIBERSORT algorism, the infiltration of 22 different immune cells was evaluated for each GC patients [26]. To assess the relationship between FRLSG and immune cell infiltration, the Wilcox Test correlation analyses were conducted between the high- and low-FRLSG groups. Furthermore, Single sample Gene Set Enrichment Analysis (ssGESA) and The Estimation of STromal and Immune cells in MAlignant Tumors using Expression data (ESTIMATE) algorithm were performed to evaluate the enrichment level of 24 kinds of immune cells [27] and the immune infiltration score for each GC patient using the “GSVA” and “estimate” R package, respectively [28, 29].

### Drug sensitivity analysis

Using the R package “pRRophetic”, transcriptome data of hundreds of cancer cell lines and drug sensitivity data obtained from three large pharmacogenomic projects, including the Cancer Therapeutics Response Portal (CTRP), Repurposing dataset PRISM (PRISM) and the Genomics of Drug Sensitivity in Cancer (GDSC) were integrated to predict transcriptome data-based drug response with the ridge regression model [30]. Moreover, the efficacy of immunotherapy for two groups was predicted based on the available data from melanoma patients who received anti-programmed cell death protein-1 (PD-1) inhibitor and anti-cytotoxic T-lymphocyte-associated protein-4 (CTLA4) inhibitor by the module named “SubMap” in GenePattern [31].

### Cell culture and quantitative real-time PCR

The human gastric epithelial cell (GES-1) and GC cell lines (AGS and HGC27) were purchased from the Shanghai Institute of Biochemistry and Cell Biology, Chinese Academy of Sciences, Shanghai, China. All cell lines were cultured in RPMI-1640 medium (Gibco BRL, Grand Island, NY, USA) supplemented with 10% fetal bovine serum (Invitrogen, Camarillo, CA, USA) and 1% penicillin/streptomycin (BasalMedia), at 37°C in a 5% CO2 atmosphere.

Total RNA from GC cells was isolated with TRIZOL reagent (EZBioscience, B004DP) according to the manufacturer protocol. The RNA samples were reversely transcribed to extract corresponding cDNA using Reverse Transcription Kit (EZBioscience, A0010CGQ), and the Quantitative Real-time PCR (qPCR) was performed using SYBR^®^ Green qPCR Master Mix (EZBioscience, A0012-R2). The amplification protocol was as follows: 95°C for 10 mins followed by 40 cycles of 95°C for 15 s and 60°C for 30 s. The internal reference 18s rRNA was utilized as an endogenous control to normalize the expression of each target lncRNAs, and the relative expression quantity was calculated by the following formula: 2 −ΔΔCt (ΔCt = ΔCt target –ΔCt β-actin). Indicated lncRNA expression was measured by qPCR methods with the LightCycler^®^ 480 System (Roche, Basel, Switzerland). Sequences of primers and 18s rRNA were listed as follows:

AL049840.4: F:AAAACAGACGCCGAGGTGAT; R:ACATGACAGTGGCAAGCTGA

AC005532.1: F:GAGTGGGGAGTTCTTGGGAA; R:GGCCACAGATAACTGCTGCT

AC004816.1: F:CGCCTGGTTGCAGAGTGA; R:CTGGACGGAAAGGCTTGGAC

LINC01357: F:CAGTTCAGTGACCTCGGGAA; R:GGCAAGTTGCATGGGTTCTC

AL355574.1: F:TGCTTTCCTCAGGCTCTAAGG; R:CCTGTCCACCTCGTGTTCTT

18 s rRNA: F:GTAACCCGTTGAACCCCATT; R:CCATCCAATCGGTAGTAGCG

### Comparison between FRLSG and previously reported ferroptosis-related lncRNAs signatures

To verify the prognosis prediction ability of FRLSG, the 1 year, 3 years, and 5 years ROC values of FRLSG were compared with four previously published ferroptosis-related lncRNAs signatures for GC, including Wei signature [32], Pan signature [33], Zhang signature [34], and Chen signature [35].

### Statistical analysis

Unless otherwise specified, all statistical analyses were conducted with R software (version 4.1.3). *P*-value < 0.05 was considered statistically significant.

### Data availability

All data are publicly available. The transcriptome data in this paper are derived from TCGA database (http://www.tcga.org).

## RESULTS

### Identification of FRLs

Annotation files from the “GENCODE” website were utilized to identify lncRNAs, and 15074 lncRNAs were obtained. Differential expression analysis was performed between tumor tissue and normal tissue, and 326 differentially expressed lncRNAs were finally selected. A total of 259 FRGs (Driver: 108; marker: 111; suppressor: 69) were extracted from the FerrDb (Supplementary Table 1). Moreover, Pearson correlation analysis was carried out between differentially expressed lncRNAs and FRGs, and 296 FRLs were identified (Supplementary Table 2).

### Establishment of FRLSG

**Figure 1:**
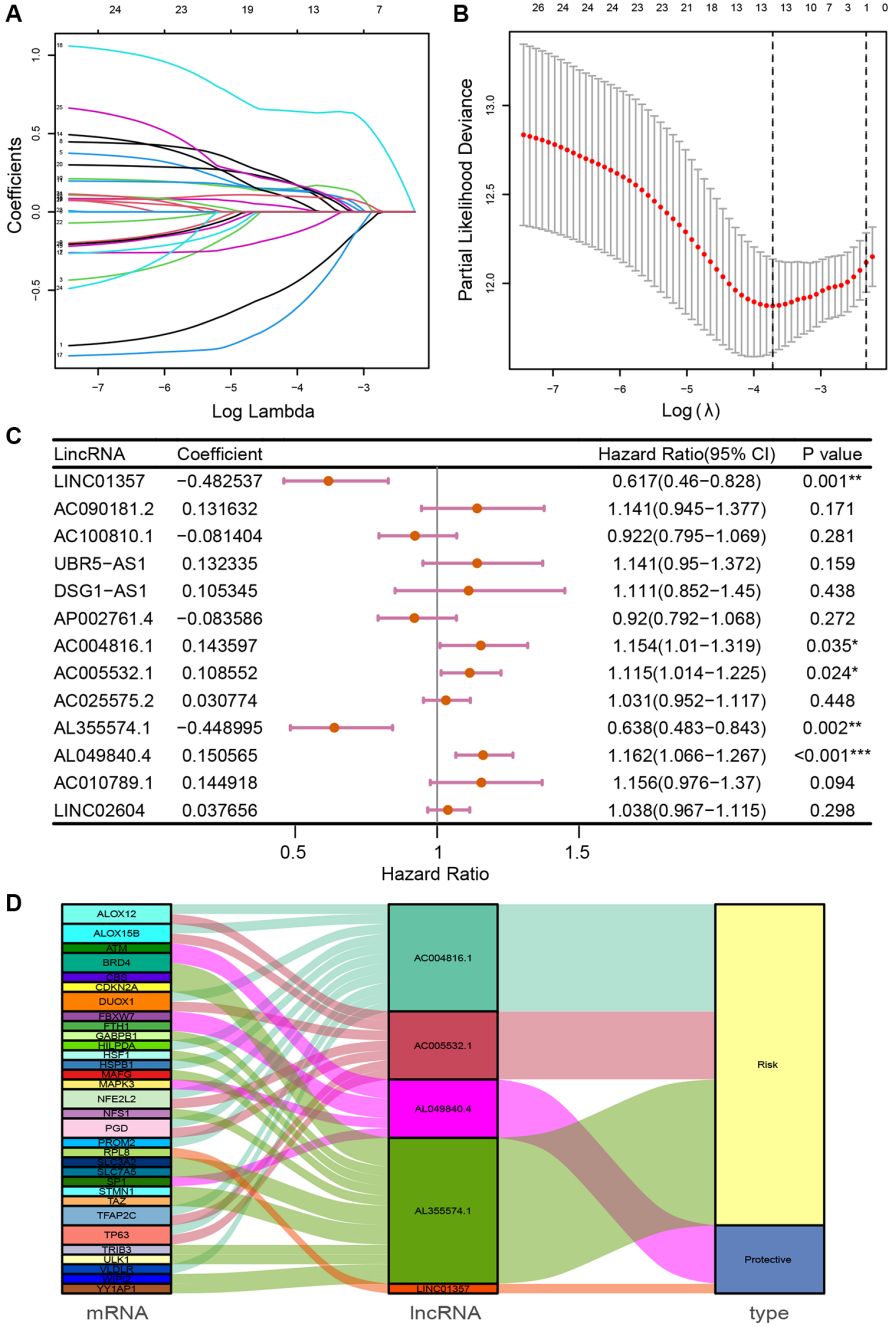
**Identification of prognosis-related FRLs and establishment of FRLSG.** (**A**, **B**) The LASSO regression model for the identification of the most robust FRLs. (**C**) Forest plot demonstrating FRLs associated with GC survival, analyzed by multivariate Cox regression. ^*^P < 0.05, ^**^P < 0.01, and ^***^P < 0.001. (**D**) Sankey diagram showing the relationships among FRGs, FRLs and risk type.

Univariate Cox regression analysis identified 28 prognosis-associated FRLs, which were included into the LASSO regression analysis (Figure 1A, 1B). 5 FRLs (LINC01357, AC004816.1, AC005532.1, AL049840.4 and AL355574.1) were identified for the construction of the risk model using multivariate Cox regression analysis (Figure 1C). Sankey diagram was utilized to demonstrate the relationships among mRNA, FRLs and risk type (Figure 1D). The formula of FRLSG was as follows: FRLSG = (−0.482537 × expression_LINC01357_) +  (0.143597 ×  expression_AC004816.1_) + (0.108552 × expression_AC005532.1_) +  (−0.448995 × expression_AL049840.4_) + (0.150565 × expression_AL355574.1_).

### FRLSG shows great prognostic prediction ability

**Figure 2:**
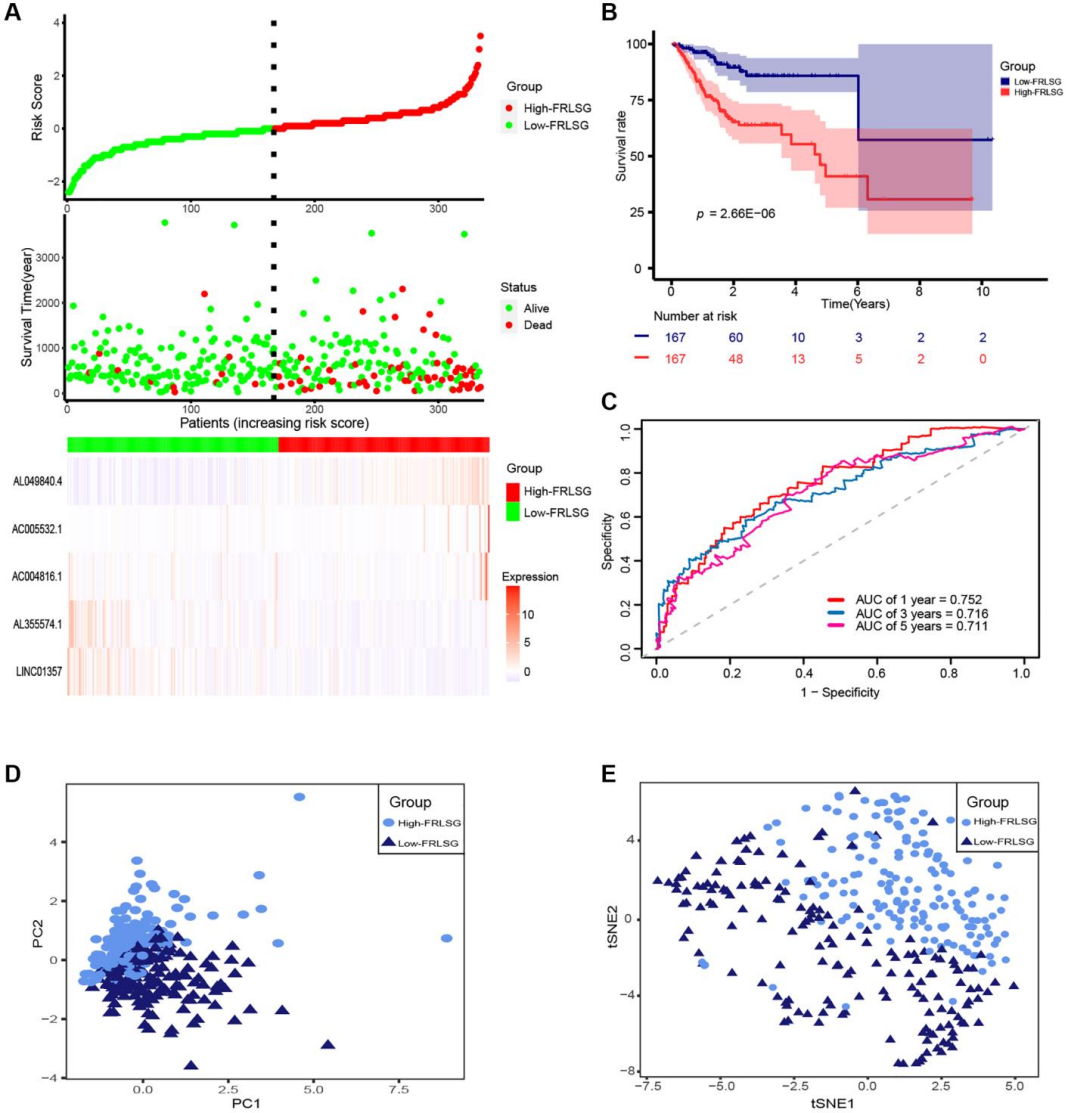
**Evaluation of prognosis predicting power for FRLSG.** (**A**) Risk survival status plot (FRLSG score distribution, scatter plots for survival status, and heatmap displaying the expression of 5 FRLs). (**B**) Kaplan-Meier curve showing the difference of prognosis between the high- and low-FRLSG groups. (**C**) ROC curves demonstrating the predicting power of FRLSG in predicting 1/3/5-year survival for GC patients. (**D**) PCA plot and (**E**) t-SNE plot for the clustering of GC patients based on 5 FRLs.

The patient risk survival status plot showed FRLSG was proportional to the number of deceased GC patients (Figure 2A). Kaplan–Meier curve indicated that patients in the high-FRLSG group had worse survival in comparison with those of the low-FRLSG group (*P* = 2.66E-6, Figure 2B). ROC analysis was utilized to evaluate the prognostic accuracy of FRLSG, and the area under curve (AUC) showed that FRLSG had a reliable ability in predicting survival times for GC patients (1 year AUC = 0.752, 3 years AUC = 0.716, 5 years AUC = 0.711; Figure 2C). In addition, the result of PCA and t-SNE showed distinct division between the high- and low-FRLSG groups based on 5 FRLs (Figure 2D, 2E).

### FRLSG was an independent prognostic factor for GC patients

**Figure 3:**
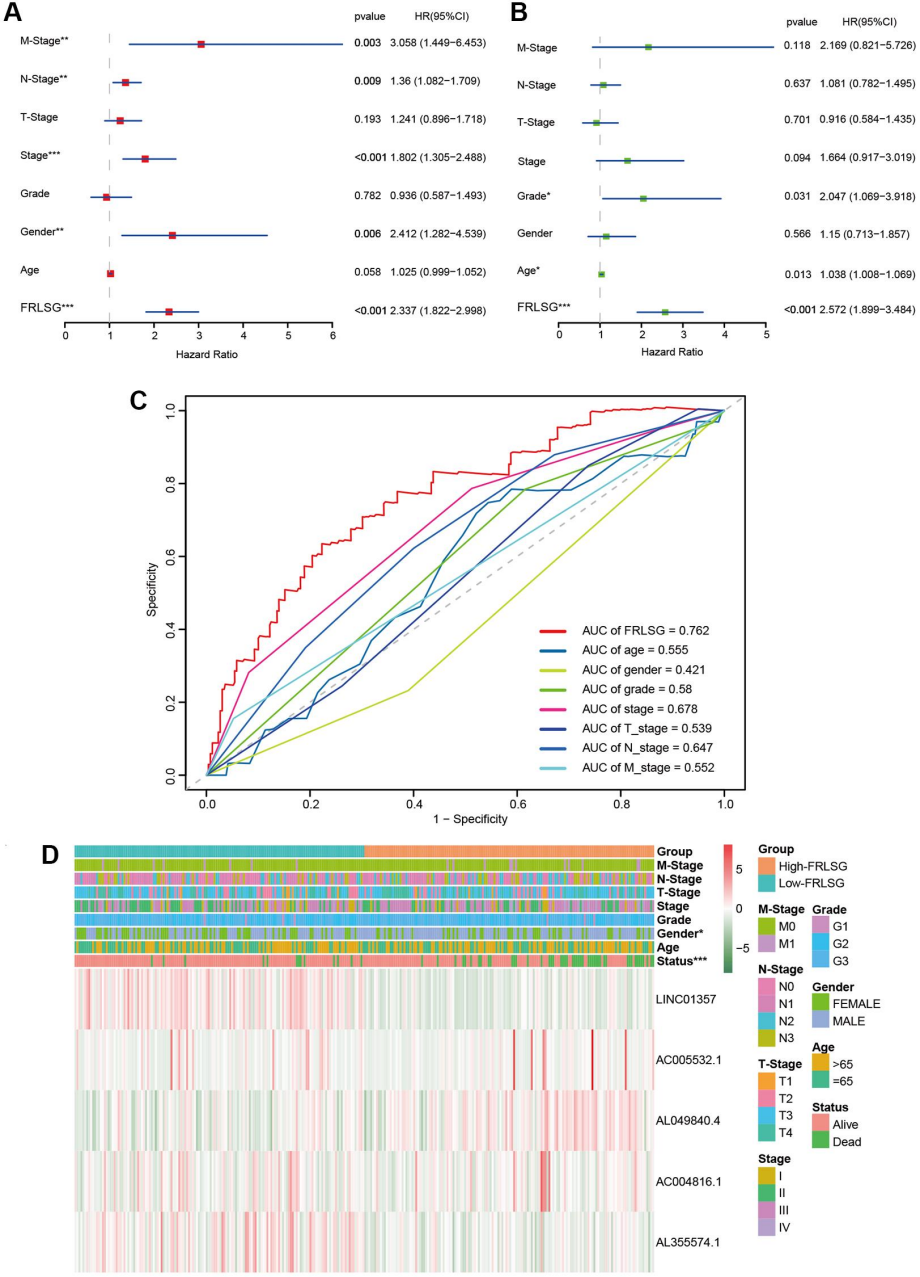
**Comparison between FRLSG and clinicopathological characteristics in prognosis predicting power.** (**A**, **B**) Forest plots demonstrating the FRLSG was an independent prognostic factor for GC. (**C**) Multi-parameter ROC curve showing the AUC of FRLSG (0.762) was higher than other clinicopathological manifestations. (**D**) Heatmap showing the clinicopathological characteristics and expression of 5 FRLs in the high- and low-FRLSG groups. ^*^P < 0.05, ^**^P < 0.01, and ^***^P < 0.001.

Univariate analysis showed that FRLSG (*P* < 0.001), gender (*P* = 0.013), stage (*P* < 0.001), N-Stage (*P* = 0.009) and M-Stage (*P* = 0.003) were independent risk factors for GC patients (Figure 3A). In addition, multivariate analysis further proved that FRLSG (HR = 2.572, 95% CI = 1.899–3.484, *P* < 0.001), age (HR = 1.038, 95% CI = 1.008–1.069, *P* = 0.013) and grade (HR = 2.047, 95% CI = 1.069–3.918, *P* = 0.031) were independent prognostic indicators for GC patients (Figure 3B). Meanwhile, Multi-parameter ROC curve revealed that the AUC value of FRLSG (0.762) was significantly higher than other clinical indicators, indicating that FRLSG had better ability in prognostic prediction (Figure 3C). Furthermore, the heatmap demonstrated the expression of 5 FRLs in the high- and low-FRLSG groups (Figure 3D). Notably, the FRLSG was proved to be tightly associated with gender based on Fisher’s exact probability test (Figure 3D).

### The FRLSG-integrated nomogram further enhances prognostic prediction power

**Figure 4:**
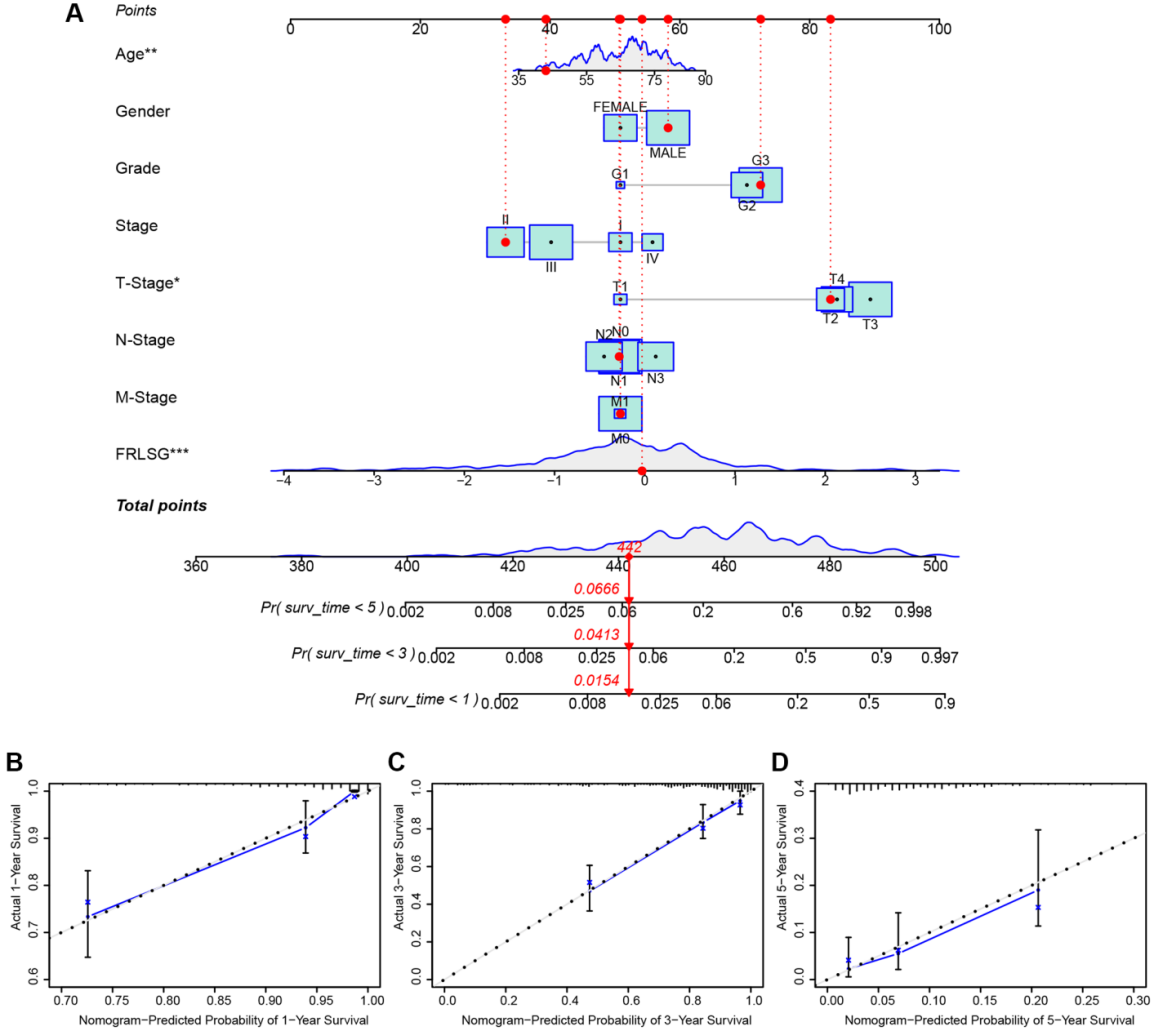
**FRLSG-integrated nomogram improving the prognostic prediction power.** (**A**) nomogram plot based on clinic-pathological factors and FRLSG (**B**–**D**) Calibration curves for the predictive accuracy (1-, 3-, and 5-year survival) of the FRLSG-integrated nomogram.

Several clinic-pathological factors and FRLSG were used to construct a hybrid nomogram to improve the prognostic prediction power (Figure 4A). The total points of all factors for each patient could be calculated according to the nomogram, which might provide a novel quantitative tool for clinical practice. The calibration curves showed good performance for the nomogram, suggesting that the nomogram was accurate and reliable in predicting the prognosis for patients with GC (Figure 4B–4D).

### Enrichment analyses and immune infiltration analysis demonstrate high immune infiltration status of patients in the high-FRLSG group

**Figure 5:**
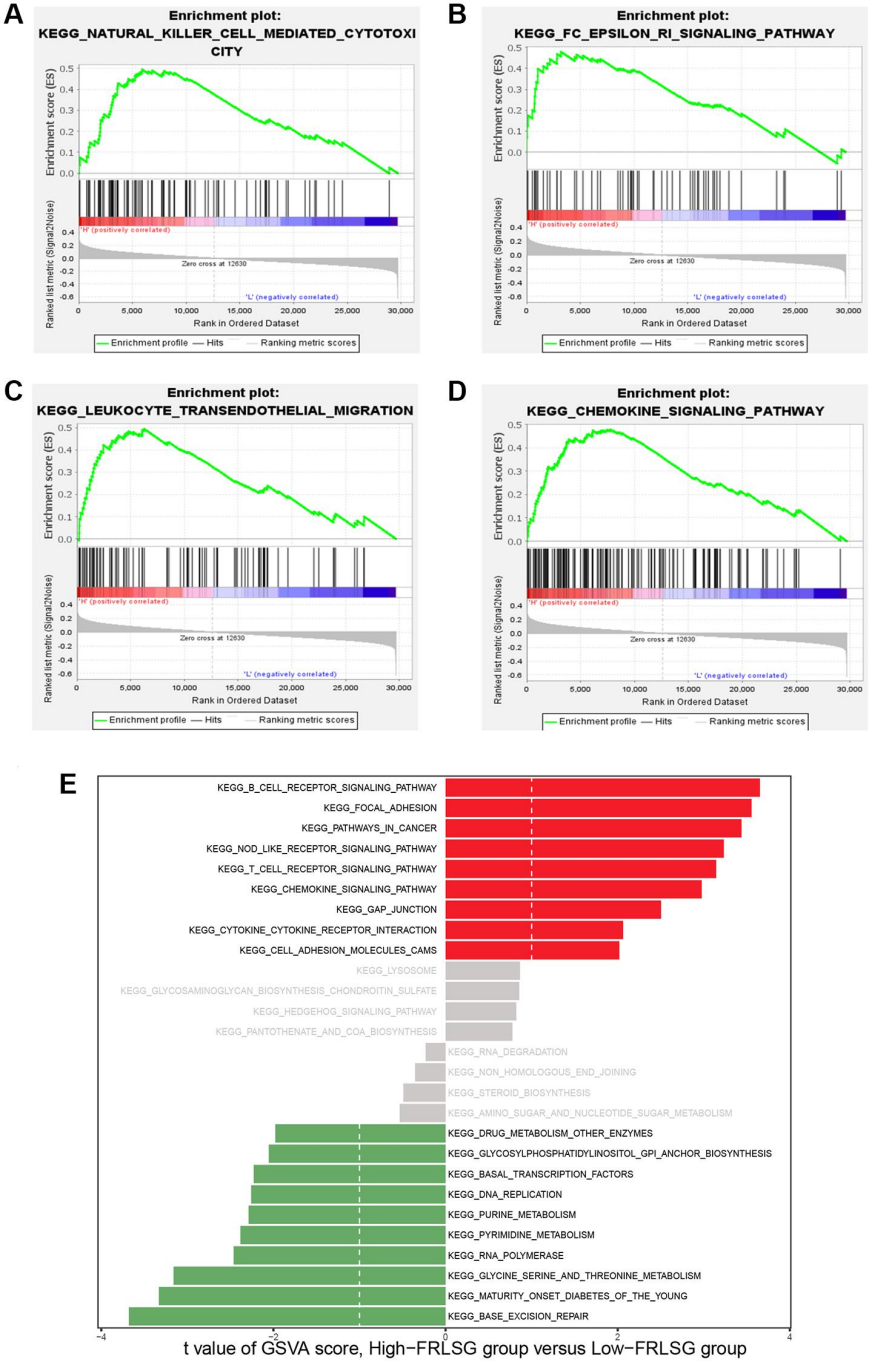
**GSEA and GSVA demonstrating the immune-related pathways enriched in the high-FRLSG group.** (**A**–**D**) Immune-related pathways enriched in the high-FRLSG group through GSEA. (**E**) GSVA demonstrating the enriched pathways associated with FRLSG. Blue bars indicated the high-FRLSG group associated pathway, while green bars indicated the low-FRLSG group associated pathways.

To investigate the pathways associated with FRLSG, we employed GSEA software to conduct KEGG enrichment analysis between the high- and the low-FRLSG groups. The result revealed that a total of 17 pathways (Supplementary Table 3) were significantly enriched in the high-FRLSG group. To our surprise, KEGG analysis demonstrated several immune-related pathways such as natural killer cell mediated cytotoxicity, Fc epsilon RI signaling pathway, leukocyte transendothelial migration and chemokine signaling pathway were enriched in the high-FRLSG group, indicating high immune infiltration status of GC patients with high-FRLSG (Figure 5A–5D). Furthermore, GSVA analysis showed that the high-FRLSG group enriched in many immune-related pathways, such as B cell receptor signaling and chemokine signaling, and CTLA4 associated pathways, such as cell adhesion molecules cams and T cell receptor signaling, which suggested GC patients with high-FRLSG might benefit from immunotherapy and anti-CTLA4 therapy (Figure 5E).

**Figure 6:**
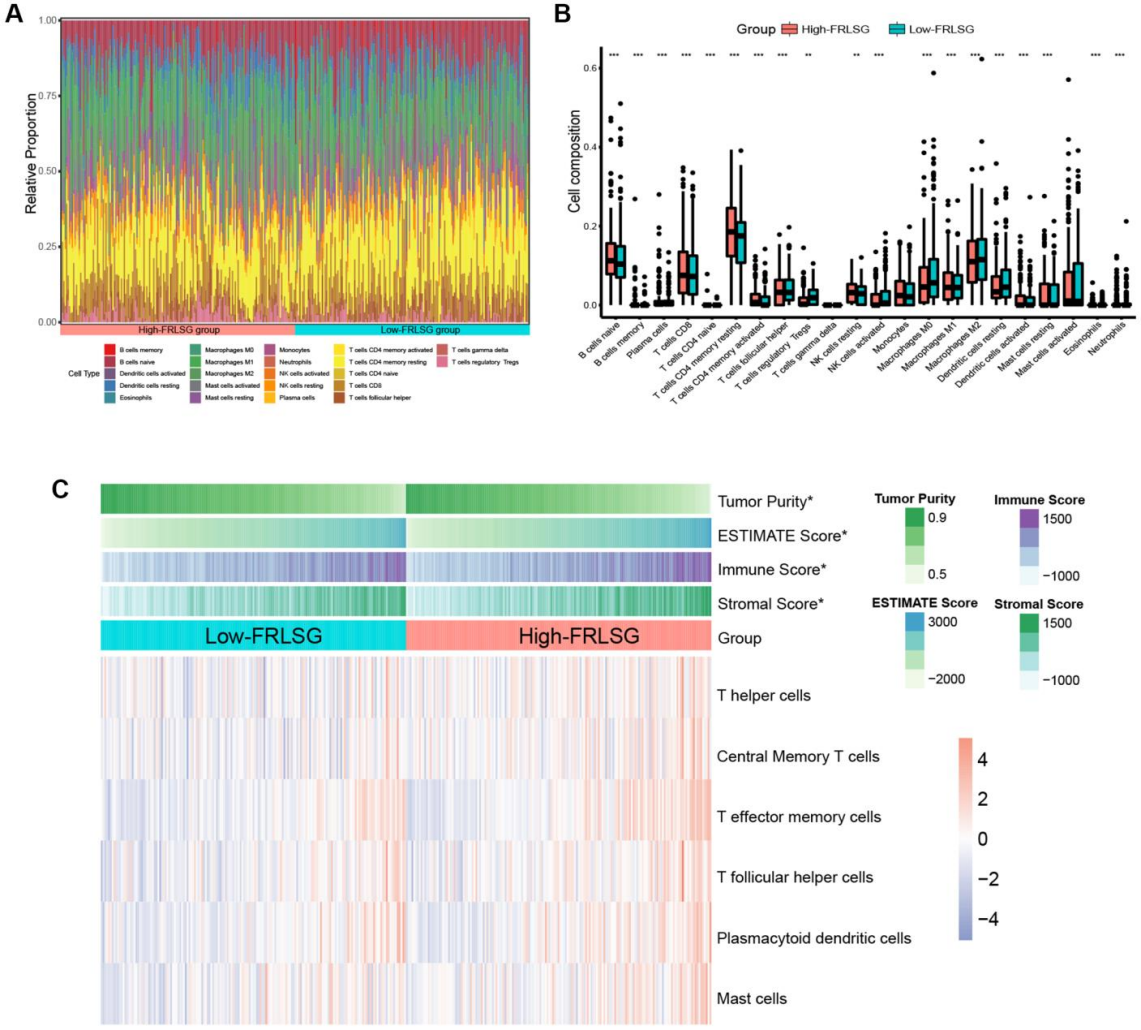
**Immune infiltration analyses indicating the high immune infiltration status of high-FRLSG patients.** (**A**) Stacked histogram demonstrated 22 different specific immune cells infiltration in each GC patient. (**B**) boxplot showed the difference of immune cell infiltration between the high- and low-FRLSG patients. ^*^P < 0.05, ^**^P < 0.01, and ^***^P < 0.001. (**C**) Heatmap illustrated the estimated scores of immune signatures calculated by ssGSEA and ESTIMATE algorism in the high- and low-FRLSG patients.

We further constructed an immune landscape to analyze the 22 kinds of immune cells infiltration in GC patients (Figure 6A). Except for monocytes, gamma delta T cells and mast cell activated, 19 kinds of the immune cells showed significant difference between the high- and low-FRLSG groups. Compared with the low-FRLSG group, the high-FRLSG group had a higher percentage of 14 kinds of immune cells, such as CD8+ T cells, naive CD4+ T cells and activated memory CD4+ T cells, which was consistent with the results of enrichment analyses (Figure 6B). The result of ESTIMATE algorism showed that GC patients with high-FRLSG tended to exhibit high immune score and ESTIMATE score (Figure 6C). In addition, ssGSEA indicated that T helper cells, central memory T cells, T effector memory cells, T follicular helper cells, plasmacytoid dendritic cells and mast cells were evaluated in high-FRLSG patients, which further validated the high immune infiltration status of high-FRLSG patients (Figure 6C).

### Distinct sensitivity to immunotherapy and targeted therapies prediction

**Figure 7:**
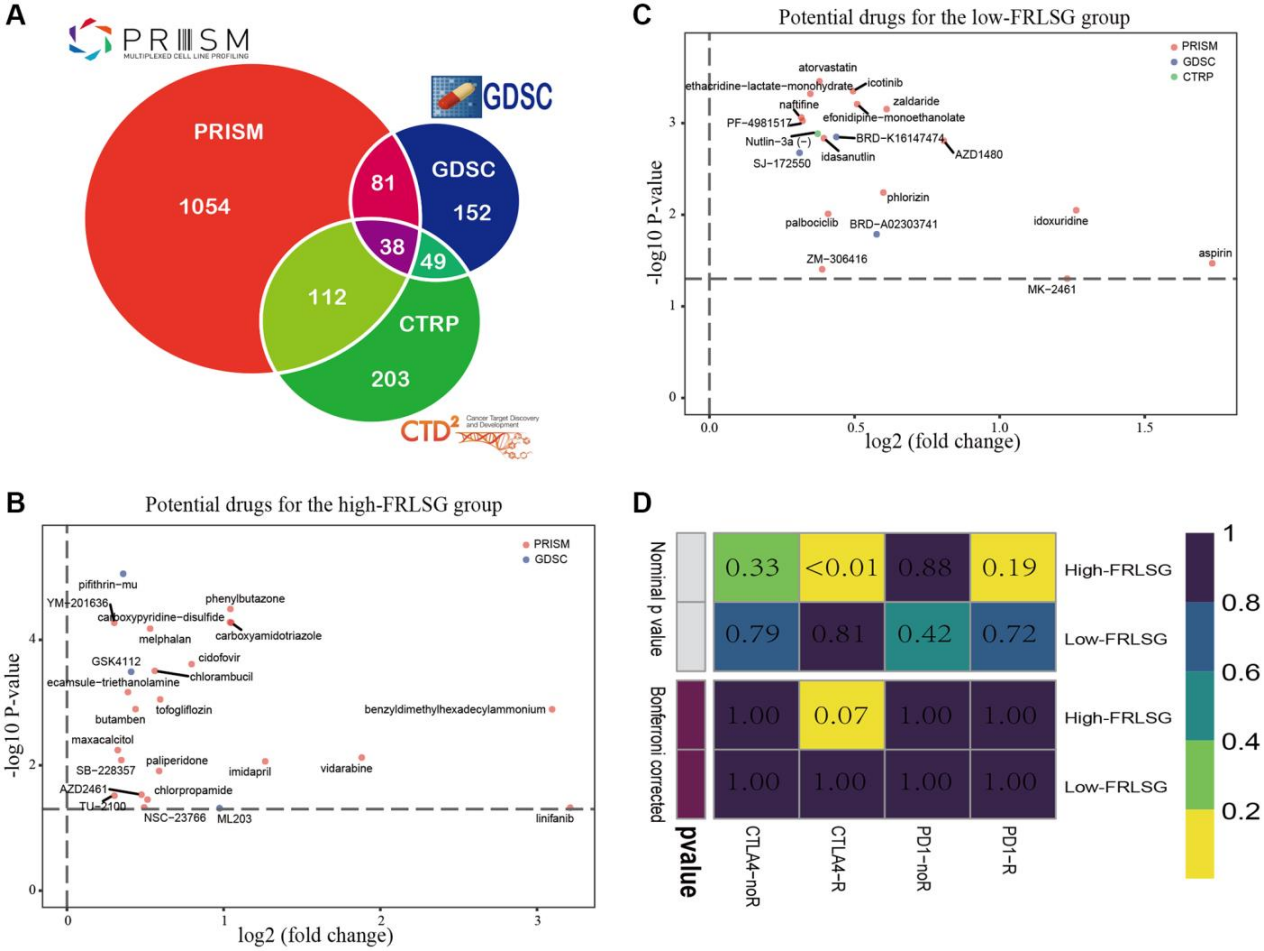
**Drug prediction and immunotherapy prediction uncover potential clinical treatment associated with FRLSG.** (**A**) Venn plot for summarizing available compounds in CTRP, GDSC and PRISM projects. (**B**) Scatter diagram demonstrating candidate drugs with potential therapeutic effect for (**B**) high-FRLSG patients and (**C**) low-FRLSG patients (**D**) Prediction of the response to anti-PD-1 therapy and anti-CTLA4 therapy for the high- and low-FRLSG GC patients.

Three pharmacogenomic datasets (CTRP, GDSC and PRISM) containing drug response data and transcriptome profiles of multiple cancer cell lines were used to establish prediction model of drug response. The Venn plot showed the number of drugs of the datasets mentioned above and their intersection (Figure 7A). Differential analysis was performed on computed drug response data between the high- and low-FRLSG patients, and compounds with lower estimated AUC values (log_2_FC > 0.30, *P* < 0.05) were considered to be effective. 19 kinds of compounds (3 CTRP-derived compounds, 1 GDSC-derived compounds and 15 PRISM-derived compound) were identified to be potential targeted therapies for the high-FRLSG group (Figure 7B, Supplementary Table 4). Some of these compounds, such as atorvastatin [36] and Palbociclib [37], had been proved to be able to suppress the proliferation and migration of GC cells, which further validated the candidate drug prediction. Besides, GC patients with low FRLSG might be sensitive to 25 different compounds (Supplementary Table 5), which were presented in a scatter plot (Figure 7C).

Using subclass mapping, we compared the transcriptome profiles of two GC subclasses separated by FRLSG with another published dataset containing 47 patients with melanoma who received anti-PD-1 and anti-CTLA4 immunotherapy treatment. The result of SubMap analysis suggested that the high-FRLSG patients had higher drug sensitivity to anti-CTLA4 therapy (*p* < 0.05), which was consistent with the immune- and CTLA4-related pathways enriched in the high-FRLSG group (Figure 7D).

### GC cell lines showing high expression of 5 FRLs

**Figure 8:**
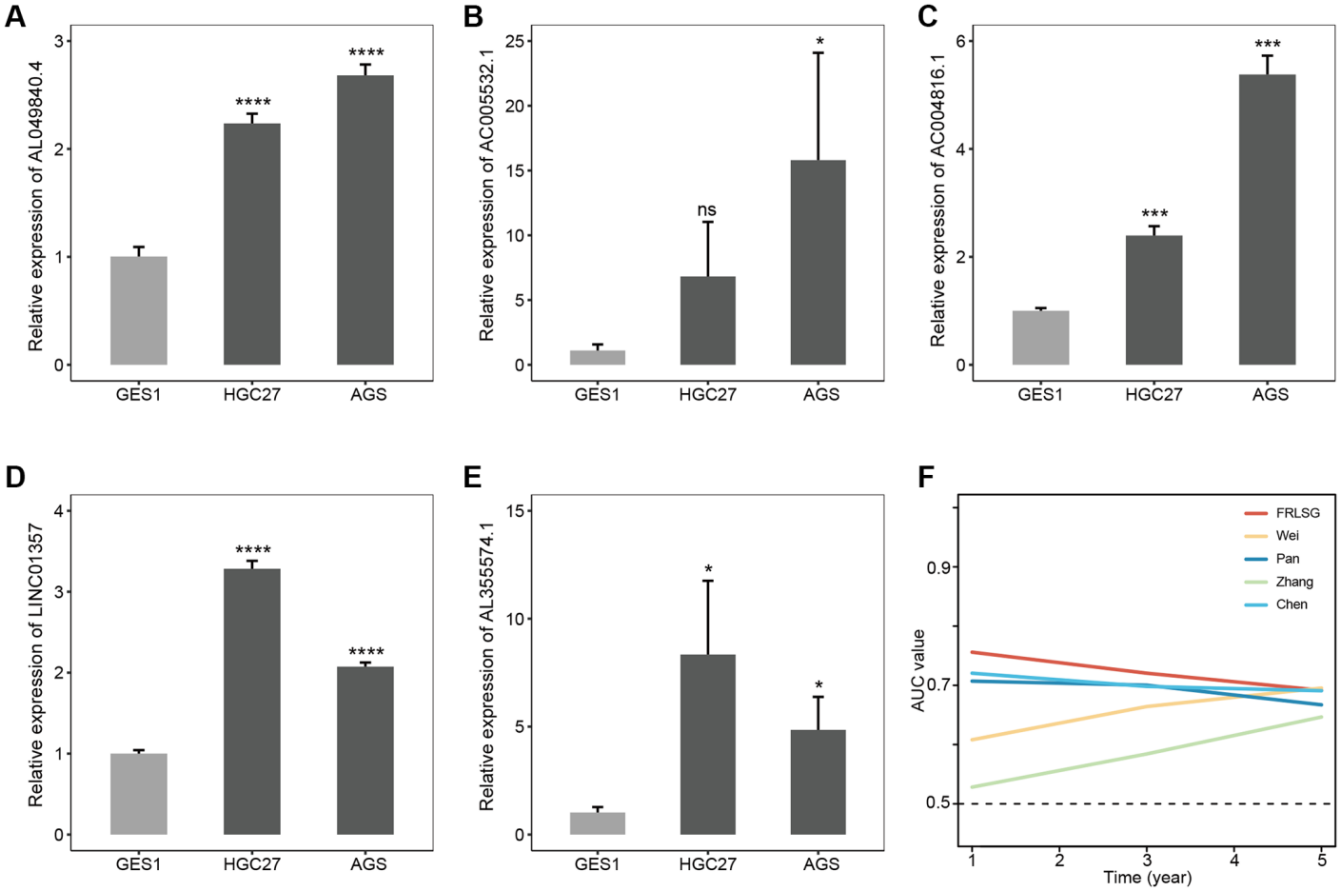
**Experimental validation and parallel comparison of FRLSG.** The expression level of (**A**) AL049840.4, (**B**) AC005532.1, (**C**) AC004816.1, (**D**) LINC01357, (**E**) AL355574.1 in the GES1, HGC27 and AGS. ^*^P < 0.05, ^**^P < 0.01, ^***^P < 0.001, and ^****^P < 0.0001. (**F**) Time-dependent AUC for the comparison of FRLSG with other four previously published ferroptosis-related lncRNAs signatures for GC.

The result of qRT-PCR assay showed AC004816.1, AC005532.1, LINC01357, AL355574.1 and AL049840.4 were overexpressed in HGC27 and AGS compared with GES1, which was consistent with the bioinformatic analyses in our study (Figure 8A–8E). In addition, compared with other four published ferroptosis-related lncRNAs signatures for GC patients, FRLSG demonstrated the highest AUC value, suggesting the priority of FRLSG in prognosis prediction (Figure 8F).

## DISCUSSION

The 5-year survival rate of GC ranges from 5% to 69%, which attributes to complex disease heterogeneity, late diagnosis, and suboptimal therapies [38, 39]. With the development of high throughput sequencing, multiple molecular biomarkers are proposed to assess the prognosis and choose therapies for GC patients, which even be more effective than TNM staging and histopathological diagnosis to some extent [40, 41]. However, to date, some molecular signatures are proved to be not stable and universal due to the heterogeneity of GC patients [42]. Therefore, a reliable and applicable signature is needed for customizing the diagnoses and treatments of GC patients.

Compared with protein-coding genes, lncRNAs are proved to regulate fewer targets through simpler pathways [43]. Recently, several studies show that lncRNAs have critical functions in regulating diverse biological processes in GC [44]. Chen et al. [45] reported that lncRNA-SNHG15 could promote proliferation, migration, invasion and apoptosis of GC cells by regulating the expression of miR-506-5p. Overexpression of lncRNA-UCA1 could down-regulate the expression of PD-L1 via repressing the expression of miR-193a and miR-214, resulting in the proliferation, distant migration and immune evasion of GC cells [46]. Further, lncRNA-DLEU2 activated by STAT1 could promote malignant progression of GC through modulating miR-23b-3p/NOTCH2 axis and Notch signaling pathway [47]. Meanwhile, previous studies indicated that ferroptosis was closely related to GC. For example, Apatinib was proved to down-regulate the expression of GPX4 by inhibiting SREBP-1a, which could induce ferroptosis in the GC cells and contribute to the multi-drug-resistant GC cells [48].

In this study, we proposed FRLSG composed of 5 FRLs (AC004816.1, AC005532.1, LINC01357, AL355574.1 and AL049840.4). Among these 5 FRLs, AC004816.1, AC005532.1 and AL049840.4 were risk genes, while LINC01357 and AL355574.1 were protective genes. The result of qRT-PCR assay verified the high expression level of these 5 FRLs in GC cell lines, which were first reported in GC. A recent study showed that AC004816.1, as one of the immune-related lncRNAs, could guide the treatment of prostate cancer [49]. AL049840.4 was reported to be a protective factor in colorectal cancer. Moreover, Miao et al. [50] reported that a seven prognostic lncRNAs signature which contained AC005532.1 could be regarded as a potential prognostic indicator and might have significant clinical value in the treatment of oral squamous cell carcinoma. However, the other 2 lncRNAs have not been reported before and needed further research. Predictive power evaluation, including ROC curve analysis, Kaplan-Meier survival analysis, and risk score plot indicated the high specificity and sensitivity of FRLSG, which was further validated by the AUC value comparison with other previously reported ferroptosis-related lncRNAs signatures for GC. In addition, the FRLSG-integrated nomogram further improved the prognosis predictive power, which might be better applied in clinical practice.

Recently, ferroptosis in tumor tissues was reported to be tightly correlated to immune cell infiltration. Aberrant ferroptosis in tumor tissue could contribute to abnormal increase of granulocyte ratio [51]. To our surprise, the result of GSEA demonstrated several immune-associated pathways were enriched in the high-FRLSG group. Meanwhile, ferroptotic cancer cells were proved to release special signals to induce phagocytosis and promote antigen presentation of dendritic cells [52]. In this study, CIBERSORT algorism demonstrated that the high-FRLSG group had a higher percentage of 14 kinds of high immunoreactive cells, suggesting that FRLSG could reveal immune infiltration to some extent. Based on the result of SubMap analysis, we found the high-FRLSG group might be sensitive to anti-CTLA4 checkpoint inhibitor. Since the screening of the target population becomes a big challenge in immunotherapy, our study might help to screen the benefited population and prolong the prognosis of GC patients. Actually, previous researches have proved ferroptosis could enhance the effectiveness of immunotherapy [53]. For example, Jiang et al. [54] reported that the inhibition of TYRO3 induced tumor ferroptosis and made drug-resistant tumors sensitive to anti-PD-1 therapy. Wan et al. [55] found that radiation-induced bystander effect accompanied by radiotherapy could achieve broad antitumor effects and contribute to immunogenic death mainly by inducing ferroptosis. Interestingly, the microenvironment of hyper-inflamed tumors was enriched in iron, which could result in cancer development and immune escape (T cell dysfunction) [56]. Therefore, inducing ferroptosis rationally to trigger stronger immune response for anti-tumor therapy remains a significant problem to be solved. Drug sensitivity analysis uncovered 19 kinds of compounds for high-FRLSG patients, which might achieve better therapeutic effect in combination with immunotherapy. Besides, 24 kinds of compounds might help to improve the treatment of the low-FRLSG patients, and some compounds have been shown to be effective on GC cells in *in vitro* experiment. For example, GSK4112, as an agonist of Rev-erbα, was proved to be able to decrease proliferation, glycolytic flux and the pentose phosphate pathway in human GC cells.

Although FRLSG is proved to be reliable and effective, there are still two limitations. First, without external cohort verification, the universality of FRLSG needs to be further validated. Second, the underlying regulatory mechanisms of 5 lncRNAs in ferroptosis remain unclear. Therefore, comprehensive *in vivo* and *in vitro* experiments were needed to uncover more convincing evidences for the validation and development of FRLSG.

In conclusion, we proposed FRLSG composed of 5 FRLs, which demonstrated high prognosis predicting power for GC patients. FRLSG could also help the clinical decision-making of immunotherapy, offering an innovative route for the individualized treatment of GC patients.

## Supplementary Materials

Supplementary Tables 1-3

Supplementary Tables 4 and 5
