# Supplementary material for: Clinical outcomes and potential therapies prediction of subgroups based on a ferroptosis-related long non-coding RNA signature for gastric cancer
Source: Aging (Albany NY). 2022 Aug 14;14(15):6358–76. doi: 10.18632/aging.204227 (PMC9417219; doi:10.18632/aging.204227)
Supplement: Supplementary Tables 4 and 5 [file aging-14-204227-s002.pdf]

SUPPLEMENTARY TABLES

Supplementary Table 4. 19 candidate drugs with potential therapeutic effect for the high-FRLSG group (log2FC > 0.30, *P* < 0.05)

Drug	conMean	treatMean	|logFC|	*p*Value	fdr	type
idasanutlin	−0.19818	−0.1508	0.394215	0.001461	0.011861	PRISM
ethacridine-lactate-monohydrate	−0.11598	−0.09115	0.347592	0.000478	0.005531	PRISM
palbociclib	−0.10424	−0.07852	0.408806	0.009767	0.043935	PRISM
Nutlin-3a (−)	−0.07444	−0.05748	0.372918	0.001303	0.016995	GDSC
ZM-306416	−0.05614	−0.0429	0.388245	0.039363	0.117144	PRISM
efonidipine-monoethanolate	−0.04751	−0.03339	0.509001	0.00062	0.006785	PRISM
AZD1480	−0.04163	−0.02378	0.807676	0.001555	0.012392	PRISM
idoxuridine	−0.03208	−0.01336	1.263778	0.008934	0.041489	PRISM
BRD-K16147474	−0.01488	−0.01099	0.437319	0.001416	0.016616	CTRP
aspirin	−0.00987	−0.00297	1.732654	0.033874	0.107184	PRISM
SJ-172550	0.006775	0.008401	0.310414	0.002107	0.020161	CTRP
BRD-A02303741	0.006509	0.009704	0.576139	0.016328	0.07918	CTRP
PF-4981517	0.008667	0.010825	0.320779	0.000943	0.008695	PRISM
phlorizin	0.008629	0.013074	0.599344	0.005727	0.031042	PRISM
MK-2461	0.012918	0.030357	1.232687	0.049789	0.138217	PRISM
zaldaride	0.019918	0.030411	0.610559	0.000702	0.007197	PRISM
naftifine	0.034285	0.042687	0.316252	0.000866	0.008253	PRISM
icotinib	0.047077	0.066336	0.494775	0.000446	0.005378	PRISM
atorvastatin	0.135491	0.176215	0.379138	0.000349	0.00455	PRISM

Supplementary Table 5. 24 candidate drugs with potential therapeutic effect for the low-FRLSG group (log2FC > 0.30, *P* < 0.05).

Drug	conMean	treatMean	|logFC|	*p*Value	fdr	type
YM-201636	−0.085696364	−0.105619322	0.3015679	0.0000534	0.0012764	PRISM
melphalan	−0.071332115	−0.103034803	0.5305081	0.0000663	0.0014133	PRISM
carboxyamidotriazole	−0.021353797	−0.044201462	1.0496014	0.0000534	0.0012764	PRISM
butamben	−0.030141048	−0.040819796	0.4375394	0.0012729	0.010897	PRISM
pifithrin-mu	−0.02884134	−0.036983394	0.3587394	8.84E-06	0.0004471	CTRP
ecamsule-triethanolamine	−0.024491629	−0.032072554	0.3890505	0.0006824	0.0070478	PRISM
phenylbutazone	−0.013568854	−0.027957695	1.0429465	0.0000322	0.001011	PRISM
AZD2461	−0.018151236	−0.025231238	0.4751432	0.0294728	0.0983189	PRISM
imidapril	−0.009028195	−0.021697891	1.2650454	0.0086709	0.0408545	PRISM
GSK4112	−0.011703811	−0.015545986	0.4095637	0.0003231	0.0060203	CTRP
ML203	−0.00445746	−0.008752655	0.973499	0.0484834	0.1526968	CTRP
NSC-23766	−0.005026319	−0.007071592	0.4925329	0.046955	0.1329361	PRISM
linifanib	−0.000516665	−0.004786121	3.211556	0.0475868	0.1340443	PRISM

benzyldimethylhexadecylammonium	0.004743	0.0005546	3.0963415	0.001283	0.010897	PRISM
vidarabine	0.011022	0.0029918	1.8813115	0.0075319	0.0370972	PRISM
chlorpropamide	0.0204495	0.0143212	0.5139164	0.0353286	0.1099019	PRISM
tofogliflozin	0.0240155	0.0158991	0.595025	0.0008909	0.0083953	PRISM
paliperidone	0.0288484	0.0191914	0.5880289	0.0123762	0.0523858	PRISM
carboxypyridine-disulfide	0.0418193	0.0203301	1.0405544	0.0000524	0.0012764	PRISM
SB-228357	0.0405046	0.0318596	0.346357	0.0082752	0.0398629	PRISM
cidofovir	0.0558302	0.032164	0.7956002	0.0002439	0.0036104	PRISM
chlorambucil	0.0524382	0.0355497	0.560783	0.0003121	0.0042406	PRISM
maxacalcitol	0.0535193	0.0427514	0.3240862	0.0057467	0.0310417	PRISM
TU-2100	0.0629948	0.0510823	0.3024103	0.0305896	0.1002315	PRISM
